# Fertility Preservation: Current and Future Perspectives for Oncologic Patients at Risk for Iatrogenic Premature Ovarian Insufficiency

**DOI:** 10.1155/2018/6465903

**Published:** 2018-07-11

**Authors:** Sara Pinelli, Stefano Basile

**Affiliations:** Maternal and Child Health Department, Division of Obstetrics and Gynecology 2, Pisa University Hospital, Pisa, Italy

## Abstract

Progress in recent years in the efficacy of oncologic treatment and early diagnosis of cancer has determined an increase in life expectance in cancer patients. About 10% of all cancer cases affect women younger than 45 years; therefore nowadays approximately 5-6% of the population in childbearing age consists in cancer survivors. A crucial issue is the high risk of premature ovarian insufficiency due to possible gonadotoxic effects of oncologic treatments. Considering combined chemotherapy, radiation therapy, and bone marrow transplantation, this risk can reach 92-100%, depending on the age and ovarian reserve of the patient, as well as the schedule and type of therapy. International guidelines recommend addressing all the patients diagnosed with a neoplasia treatable with potentially gonadotoxic therapies to fertility preservation. Moreover, fertility preservation also seems to reserve fascinating implications for women who want to delay childbearing for social reasons or women affected with endometriosis, who could receive unexpected opportunities. At present, the most widespread techniques to preserve fertility in adult women are embryo or oocyte cryopreservation, depending on the presence of a partner or according to legislative issues, but these procedures require time for ovarian stimulation. In prepubertal patients or when there is no possibility of delaying chemotherapy, ovarian tissue cryopreservation and subsequent transplantation represent the main strategy.

## 1. Introduction

The issue of infertility epidemic is actual and troubling in most western countries as well in large part of the developed world. In fact, the decrease of young people who have to sustain elderly people is an alarming problem for those countries' economies [1]. Based on recent cancer statistics, a woman's probability of being diagnosed with an invasive cancer from birth to 49 years is 5.4% (1/18) [1].

In recent years, progress in the efficacy of oncologic treatment and in early diagnosis of cancer has determined an increase in life expectancy in cancer patients [2]. Therefore, approximately 6% of the population in childbearing age nowadays consists in cancer survivors [3].

At the same time complex ethical matters are raising in infertility centers together with the introduction of the newest fertility preservation procedures.

An unpleasant side effect of some chemo- and radiotherapy is premature ovarian insufficiency (POI) after treatment. When these treatments are combined with bone marrow transplantation, the risk of POI is even greater [4].

Particularly, some anticancer drugs can reduce the primordial follicle pool, induce ovarian atrophy and fibrosis of ovarian cortex, and damage gonadal vascularization [5]. However, it is well known that the effects of gonadotoxic drugs are not always the same, depending on type and dose of drug and on age and pretreatment ovarian reserve of the patient [4]. The effect can range from complete premature ovarian insufficiency (POI) to partial reduction of ovarian reserve.

## 2. Cancer Patients That Could Benefit from the Ovarian Tissue Cryopreservation

The American Society of Clinical Oncology (ASCO) in 2006 suggested the stratification of patients in high, intermediate, and low risk of ovarian insufficiency subsequent to oncologic treatments, depending on treatment regimen [6].

For example, standard first line treatment of Hodgkin lymphoma (HL) is ABVD (Adriamycin, bleomycin, vinblastine, and dacarbazine), which is considered to have a low risk of POI in reproductive age (< 10 %) [7].

On the contrary, patients undergoing regimens containing alkylating agents or hematopoietic stem cell transplant (HSCT) are strongly advised to consider fertility cryopreservation, having a POI rate of 70-100 %. The chances of becoming pregnant after these treatments are as low as 3-8 % [8].

Also, ionizing radiation on the pelvis can cause dose-dependent damage to DNA with estimated survival of only 50% of primordial follicles following a dose of <2 Gy. A dose of 5-10 Gy is considered toxic for oocytes [9].

## 3. Ovarian Reserve

It is crucial to give each patient adequate counselling regarding her risk of developing POI after oncologic treatment, but it is extremely difficult to forecast an exact percentage of risk.

The concept of “ovarian reserve” refers to the pool of primordial follicles contained in both ovaries at each stage of a woman's life and depends on the quantity of primordial germ cells that migrate in the ovaries during fetal development. Already during gestation, the great majority of these germ cells are lost before birth, and the process of depletion continues until puberty, when a woman owns around 300.000-400.000 primordial follicles in both ovaries, but this number varies greatly from one woman to another, depending on causative factors only partially elucidated [10–12].

After menarche, each month a woman loses a variable number of follicles in the process of selection of dominant follicle, so the decline in the follicular pool continues until menopause.

First, the age of the patient is decisive, as after age 35-38 years the ovarian reserve is consistently reduced. Beyond the number of remaining oocytes in the ovary, another crucial factor depending on patient's age is oocyte quality, which may influence the prognosis of therapeutic strategies for fertility preservation [11]. Moreover, each patient has a different ovarian reserve depending on genetic and environmental factors, so it is mandatory to evaluate pretreatment ovarian reserve analyzing antimullerian hormone (AMH), antral follicle count (AFC) through ultrasound (US), and, when possible, follicle-stimulating hormone (FSH) and estradiol on second-third day of menstrual cycle [13, 14].

Level of circulating AMH seems to be the most reliable parameter of ovarian reserve, as it does not fluctuate among various cycles and does not vary among different phases of menstrual cycles.

However, technical issues such as improper storage and handling of samples, delayed centrifugation, storage at room temperature can drastically interfere with the correct evaluation of AMH levels. Standardization of measurement method of AMH combined with a stable automated assay is likely to improve its performance in predicting the ovarian reserve [15].

As levels of AMH decrease over the course of cycles of chemotherapy, it could be a valid marker of gonadotoxicity of anticancer treatments [16, 17].

The proper integration of AMH levels and AFC is desirable to have a correct evaluation of the ovarian reserve before the selection of fertility preservation technique.

## 4. Options for Fertility Preservation (Table 1)

### 4.1. Gonadotropin-Releasing Hormone (Gn-RH) Agonists

The cotreatment of cancer patients with Gn-RH agonist during oncologic therapies has been used for decades. The rationale of this procedure is the evidence that maintenance of ovarian function after this treatment is more frequent in girls treated before puberty, than in women treated after menarche [18]. However, the efficacy of this strategy of fertility preservation is little discussed.

Meta-analyses conducted so far have shown that cotreatment with Gn-RH analogues may give a higher chance of restoration of menstrual cycle after cancer treatment but probably does not reduce gonadotoxicity of oncologic treatments, as it does not seem to improve the clinical pregnancy rate and the fertility potential by resuming spontaneous ovulation [19, 20].

A recent meta-analysis by Elgindy and colleagues, in 2015, examined 10 trials published on 907 women to evaluate whether Gn-RH analogues coadministration during chemotherapy can reduce ovarian gonadotoxicity [20]. Researchers concluded that Gn-RH agonists cotreatment did not significantly increase ovarian function resumption, nor any parameter of evaluation of ovarian reserve. Also, spontaneous pregnancy rates were comparable between patients who underwent cotreatment and women who were submitted to chemotherapy alone.

A long-term analysis published in 2016 evaluating 67 patients affected by lymphoma compared the fertility outcome of patients cotreated with the Gn-RH agonist Triptorelin versus patients who underwent chemotherapy alone. Multivariate logistic regression analysis showed that there was a significant association between risk of POI and age, the conditioning regimen for hematopoietic stem cell transplant, and the total dose of cyclophosphamide but that the coadministration of Gn-RH agonist during chemotherapy did not significantly affect ovarian reserve posttreatment and pregnancy rate [21].

Although the ASCO Clinical Practice guidelines suggest that cotreatment with Gn-RH analogues may have some medical benefits such as the reduction of abnormal vaginal bleeding in patients with low platelet count consequent to chemotherapy, they state that there is insufficient evidence that Gn-RH analogues are a reliable method to preserve fertility, in view of the cost and potential relevant side effects such as bone loss, hot flushes, and possible interference with response to cancer treatment in estrogen-sensitive cancer [22].

### 4.2. Ovarian Transposition (Oophoropexy)

Oophoropexy is a procedure which consists in ovarian transposition out of the field of radiation in patients undergoing pelvic or lower abdomen radiation as only cancer treatment. This technique seems to be underused, despite it being a relatively simple and quick laparoscopic surgical procedure that does not require delaying radiotherapy [4, 23].

Indications for the technique are gynecological cancer such as cervical, vaginal, and uterine carcinomas or ovarian dysgerminoma. Other possible indications are nongynecological cancers like osteosarcoma, rhabdomyosarcoma, Hodgkin's lymphoma, anorectal cancer, and medulloblastoma [24, 25].

The rates of fertility preservation vary depending on the regimen of radiation used: 90% of patients who underwent vaginal brachytherapy and 60% of patients who underwent external radiation therapy with vaginal brachytherapy after surgery had their ovarian function preserved [4, 24].

During ovarian transposition, the ovaries are mobilized with their blood supply from the infundibulopelvic ligament and then fixed with permanent suture to the anterior abdominal wall, in a lateral position, 3–4 cm above the umbilical line [23, 24]. Metallic clips are usually positioned at the level of sutures, to identify the ovaries when planning radiotherapy.

However, the technique is not always successful, because of radiation scatter. Especially in women older than 40 years, ovarian reserve is too scarce to be preserved, even with oophoropexy. Moreover, sometimes adjuvant chemotherapy can be necessary, and in these cases the risk of POI cannot be avoided by ovarian transposition alone [22]. In view of the above, during laparoscopy it could be advisable to take ovarian biopsies for ovarian tissue cryopreservation [4]. It should be remembered that this surgical procedure requires a general anesthesia and that surgery can always have the described complications of laparoscopy.

A further limitation of the technique is the theoretical possibility of havng ovarian localization of the primary tumor, so it is mandatory to inform patients of this risk and to exclude tumors with a high risk of ovarian metastases from this procedure. In view of the above-mentioned limitations, ovarian transposition remains experimental as a method to preserve fertility.

### 4.3. Embryo Cryopreservation

At present, embryo cryopreservation is the main resource for a woman at risk of iatrogenic POI who has childbearing desire [22].

The technique is well established in fertility programs, and it is used when there are supernumerary embryos in assisted reproduction technologies (ART) cycles, when there is risk of ovarian hyperstimulation syndrome, or in other circumstances.

However, the possibility is feasible only for postpubertal women with a partner (or using donor sperm), and the technique has ethical and legal issues to consider: in some countries, embryo cryopreservation is forbidden by law both in ART and fertility preservation, and embryo cryopreservation is performed notwithstanding the current regulations in very specific cases [26]. Moreover, cryopreserved embryos belong to the couple of parents, and this aspect may be problematic to manage after many years from cryopreservation, as relationships can end.

Another limit of the procedure is that it gives the woman the possibility of pregnancy but does not restore her fertility, having no effects on cycle restoration or resuming ovulation. Therefore, spontaneous conception is not possible.

Finally, the procedure is not practicable in patients who need to begin chemotherapy as soon as possible, as it requires 10-15 days for controlled ovarian stimulation (COS) to induce multiple follicular growth and oocytes pick up.

### 4.4. Oocyte Cryopreservation

In young patients with no need to urgently begin chemo/radiotherapy, oocytes cryopreservation after COS is considered a good option for future pregnancy perspectives. The recent progress in oocyte vitrification has led to results comparable to ART cycles with fresh oocytes [26–33].

Nowadays oocyte cryopreservation, together with embryo cryopreservation, is considered a well-established method for fertility preservation [22, 27]. Potential advantages of this technique also can be undertaken by single women and there is theoretically no risk of malignant cells reimplantation when it is performed in oncologic patients. Despite this, the method provides the possibility of obtaining a pregnancy but does not restore fertility of oncologic women faced with iatrogenic POI, nor spontaneous ovulation. It is not feasible for prepubertal girls, as it requires sexual maturity, nor for patients with the urgent need to undergo oncologic treatments. Indeed, two weeks is generally needed for COS. However with the background of the efficacy of the “emergency schedule” starting COS in the luteal phase, it allows maximizing the chances of success [34]. Recently a new stimulation protocol providing for a double stimulation in both follicular and luteal phase of the same menstrual cycle has been proposed with the aim of maximizing the number of oocytes retrieved in a single cycle [35]. However, women should be counselled that age at the time of fertility preservation is strongly related to the chances of success, as oocyte age determines pregnancy rate.

A recent study by Cobo retrospectively analyzed their clinical data to assess the estimated live birth rate per oocyte in women aged ≤35 and ≥36 years. The authors found a 60.5% likelihood of live birth in women ≤35 years considering a pool of 10 mature oocytes used, while the likelihood of live birth was only 29.7% for the same number of oocytes used [28]. The same relation of patient's age with chances of live birth rate after ART cycles using thawed oocytes was confirmed by Goldman and colleagues [36], who developed a model to predict patient's chances of pregnancy depending on age and the number of oocytes retrieved.

It is therefore recommended to carefully consider patient's age before submitting her to COS and pick up, informing her of the real possibility of pregnancy based on her ovarian reserve.

Another crucial aspect is the performance of the technique based on the number of oocytes retrieved. Among the most cited papers on the topic, the work of Sunkara and colleagues in 2011 showed a nonlinear relationship between the number of oocytes retrieved and the chances of pregnancy following IVF treatment [37].

Moreover, in highly qualified ART centers, the live birth rate per vitrified oocyte is reported around 5.7%, thereby approximately 10 oocytes are needed to give patient a real chance of pregnancy [3, 28–30, 36]. And it is important also not to forget that the great majority of the studies regarding the performance of thawed oocytes in ART is extrapolated by studies on infertile, nononcologic patients, but there is growing evidence that patients with certain specific cancers or patients with known mutations of BRCA gene could have a reduced ovarian reserve connected to the malignancy [38, 39]. More studies are needed to elucidate the topic.

Some authors advise against submitting to COS for oocyte or embryo cryopreservation women with hormone-sensitive cancers [40]. However, there is good evidence in literature regarding the safety of these procedure using combination protocols including Aromatase Inhibitors, such as Letrozole, associated with standard Gn-RH antagonist stimulation protocols, to avoid the supraphysiological increase in estrogens after COS [41–45]. The data in literature show comparable pregnancy rate to infertile population undergoing IVF and embryo transfer (IVF-ET) [42]. Nevertheless, concerns exist about the possible role of progesterone in hormone-dependent cancers, as the cotreatment with Letrozole has shown to not influence progesterone levels during COS in fertility preservation ART cycles [45]. A recent systematic review concludes that a detrimental effect on disease-free survival period in breast cancer women submitted to COS with Letrozole supplementation is not demonstrated in available evidence, but high-quality evidence is lacking. So, combined protocols using Letrozole 5 mg daily administrated during COS are recommended, but a slight effect on the possibility of recurrence in breast cancer is not excluded [46]. More studies on larger populations with longer follow-up periods are therefore required to clarify this topic.

### 4.5. Ovarian Tissue Cryopreservation and Grafting

Ovarian tissue cryopreservation and subsequent reimplantation are now the only available option for fertility preservation in prepubertal girls and patients who cannot delay the start of oncologic treatments. The great advantage of this method is that with grafting of thawed ovarian tissue not only pregnancy chances but also ovarian endocrine function is restored, and natural pregnancy can be achieved. When cryopreservation and storage of ovarian tissue are performed in prepubertal girls, successful subsequent grafting may also allow induced puberty [3, 47]. Regrettably, this technique presents age limitation, due to the scarcity of primordial follicles contained in the ovaries after 35-36 years [4].

Possible indications for this procedure are malignant disease or other disorders that require potentially gonadotoxic treatments, such as autoimmune diseases necessitating treatment with cyclophosphamide, severe endometriosis requiring extensive surgical eradication, genetic syndromes at risk of POI, or benign hematological diseases requiring bone marrow transplantation [40].

Unfortunately at present ovarian tissue cryopreservation and subsequent grafting are considered an experimental procedure, mainly due to the scarcity of definite data published in literature on the outcome of the technique [40, 48]. In fact, many papers have been published on successful pregnancies obtained in women after transplantation of cryopreserved tissue, but few articles report the effective total number of transplantations performed, and the majority of papers regarding this issue are case reports or case series regarding successful pregnancies, rather than failures [4, 49].

Ovarian tissue cryopreservation can be performed by laparoscopy, and 4-5 ovarian cortex slices of ≈1 cm in length, 4-5 mm in width, and 1.0-1.5 mm in depth are generally taken, reducing as far as possible the use of electrocoagulation to decrease ovarian cortex injury and consequent loss of primordial follicles [50]. In very young children, where ovarian volume is considerably lower, unilateral oophorectomy can be performed, especially when the risk of iatrogenic POI after the therapies is high [3, 51]. It is important to leave one ovary in situ, to allow future orthotopic reimplantation. Histopathologic exams can be made on one of the fragments to analyze the possible presence of malignant cells and evaluate the density of primordial follicles.

The site of subsequent grafting can be orthotopic or heterotopic. To date, the first option is preferred, firstly because it may possibly lead to spontaneous conception and because of the pelvic cavity supply with the ideal microenvironment to support the engraftment [4]. The technique of orthotopic grafting consists in the placement of the thawed ovarian tissue fragments in a “peritoneal window” of the ovarian fossa or preferably closely to the medulla of the remaining ovary, after making an incision in the cortex [47]. The ovarian slices are then fixed to their new location by interrupted sutures [3]. The technique requires abdominal surgery, usually carried out by laparoscopy, under general anesthesia. It is useful to perform laparoscopic tubal patency test during surgery, to refer patients to ART when necessary. Some authors suggest executing the procedure in two steps, by preparing a peritoneal pocket or preparing the ovarian tissue for subsequent transplantation in a first laparoscopy, to allow optimal angiogenesis to supply the graft, and then a “second step” after approximately one week [52].

Heterotopic grafting means transplantation of thawed ovarian cortex tissue outside the pelvic cavity. It can be performed in subcutaneous tissue of the abdomen or in the forearm. This method can be preferable in cases of severe pelvic adhesions or anatomic changes secondary to radiation therapy, which could impair vascularization to the graft causing ischemic injury. It does not require general anesthesia, and the graft can be easily accessible for ultrasound during COS and oocyte pick up. However, this method does not allow spontaneous conception.

Concerning the outcome of ovarian tissue cryopreservation as a fertility preservation method, some retrospective cohorts have been recently published by the most experienced research groups, with live birth rates reported around 23 to 37% [3, 51–56]. A recent meta-analysis by Pacheco and Oktay reported a cumulative clinical and live birth plus ongoing pregnancy rate of 57.5% and 37.7%, respectively, with an endocrine restoration rate of 63.9% [49]. Mean duration of ovarian function after grafting has been reported about 5-10 years, with a variability mainly due to age at the time of the cryopreservation, previous gonadotoxic treatments, and the volume of ovarian tissue removed, but it should be considered that the procedure can be repeated [52]. Unfortunately, transplanted ovarian tissue seems to undergo an initial over-recruitment of follicles and a dysfunctional folliculogenesis which determine an acute impairment of follicular pool in grafted tissue, with loss of approximately 50% of follicles, because of ischemia and oxidative stress damage [4, 57].

Some factors can thus influence the lifespan of the graft, such as the treatment with antioxidants or vascular growth factors, on which research is focusing to increase the chances of success of the technique [57–60]. In 2016, Oktay and colleagues obtained two life births in patients with iatrogenic POI after transplantation of cryopreserved ovarian tissue with a human decellularized extracellular tissue matrix scaffold, to minimize oxidative stress damage secondary to ischemia [61]. More research is needed on this topic to maximize the chance of successful growth of grafted ovarian tissue.

## 5. Risk of Reimplanting Malignant Cells with Transplanted Cryopreserved Ovarian Tissue

Ovarian tissue cryopreservation followed by orthotopic reimplantation represents the main option to preserve fertility for prepubertal girls or oncologic patients undergoing immediate gonadotoxic cancer treatment, as oocyte and embryo cryopreservation techniques require a prior course of hormonal stimulation [22].

The serious concern that remains regarding the safety of ovarian tissue preservation and subsequent grafting is the possibility of harvesting, cryopreserving, and subsequently reintroducing malignant cells present in the ovarian cortex tissue yielded. These cells in fact could lead to primary cancer recurrence within the grafted tissue [40].

According to data in literature, hematologic cancers represent the most frequent indication for ovarian tissue cryopreservation, accounting for more than 1/3 of total cases.

Hodgkin's (HL) and non-Hodgkin's lymphoma (NHL) are reported in this group of malignancies, and leukemia is particularly common in girls under the age of 20 years [4, 62].

Unfortunately, although rare, ovarian metastases have been described in a large part of malignancies, including leukemia, HL and NHL, breast cancer, renal tumors, Ewing's sarcoma, gastrointestinal system cancers, and neuroblastoma [4].

The risk of ovarian metastases from nongynecologic cancers seems higher for gastric carcinoma (55.8%), followed by colon carcinoma (26.6%), breast cancer (24.2%), lung cancer (23.4%), and lymphoma (13.3%), whereas the reported rate for uterine carcinoma ranges from 13.1% to 22%) [63]. A different issue is represented by acute leukemia that is a blood-disseminated disease and is hence considered at very high risk for ovarian metastases (> 50%) [64].

Although the relatively considerable rate of ovarian metastases reported in literature mandates concerns in grafting ovarian tissue, most data refer to research based on autopsies and therefore do not necessarily reproduce identical clinical risks especially for the women in complete disease remission and with a favorable prognosis who are offered cryopreservation of ovarian cortex.

### 5.1. Leukemia

By molecular biology studies, especially with Polymerase Chain Reaction (PCR), cancer cells have been identified in ovarian cortical samples in more than 50% of women affected by acute leukemia [3].

Apart from general data, as a matter of fact the actual risk of transferring diseased cells together with the grafted ovarian tissue is subject to the complete remission of disease, the type of leukemia, and how chemotherapy is administered [64].

Therefore, it is mandatory to establish minimal residual disease (MRD) in patients affected by hematologic diseases before planning yield and subsequent frozen-thawed reimplantation of ovarian tissue [4].

Unfortunately, evidence from literature concerning this topic is still based on research of low-moderate quality and therefore interpretation of available data must be evaluated with caution.

In consideration of the peculiarity of disease, even if further studies are needed, before cryopreservation, other options as In Vitro Maturation (IVM) [65] or isolated follicle transplantation should be considered for such women with the aim of eluding chemotherapy jeopardizing the pool of primordial follicles.

### 5.2. Hodgkin's Lymphoma

Published literature concerning the risks of transferring malignant cells in women affected by HL is as yet associated with a very low-quality evidence [4].

To date, despite occasional reports of ovarian involvement by high stage HL, mainly by autopsy studies, there is a growing testimony of the safety of ovarian tissue cryopreservation, described by several authors, with a relatively large series of 15 cases demonstrating no recurrences with a maximum follow-up of 8 years [62].

### 5.3. Non-Hodgkin's Lymphoma

All studies but two confirmed the safety of frozen ovarian tissue autotransplantation in women affected by NHL [62].

Again, low-quality evidence calls for the utmost caution when indicating fertility preservation procedures including reimplantation of cortical tissue in such patients [3, 4].

### 5.4. Breast Cancer

Since breast cancer represents the most frequent malignancy in women and about 5% of cases occurs in women under the age of 40 years, several studies have been performed to investigate risks and outcomes of harvesting and reimplanting cryopreserved ovarian cortex in patients with the disease [3].

Whereas in advanced stage patients the risk of ovarian metastases (13.2-37.8%) commands prudence in ovarian tissue autotransplantation, in early stage disease growing evidence of the safety, feasibility, and success of transferring cryopreserved ovarian cortex seems to give encouraging new horizons for such patients.

### 5.5. Cervical Carcinoma

Considering that ovarian involvement is known to be noticeably more common in adenocarcinoma (up to 6.8%) than in the usual squamous cell histotype (0.7-2.5%), no evidence of relapse from the grafted tissue has been demonstrated in all the most recent series published in literature [64].

### 5.6. Endometrial Carcinoma

The reported risk of ovarian malignant cells in women affected by endometrial cancer seems directly related to the stage of disease, being anyway very low in the frequent early stage endometrial carcinoma (1.9% in Federation of Obstetricians and Gynecologists (FIGO) stage 1 tumors) [63].

### 5.7. Central Nervous System Tumors

Primitive neuroectodermal tumors (PNETs) like medulloblastoma and neuroblastoma are classified in two types: central nervous system PNET and peripheral PNET (Ewing sarcoma).

Despite single successful reports of transplantation of frozen-thawed ovarian tissue in children, Ewing sarcoma is still considered at moderate risk for ovarian involvement, whereas neuroblastoma is categorized together with leukemia and Burkitt lymphoma in the high-risk group for ovarian metastasis by most authors [4].

### 5.8. Colorectal Carcinoma

Very few case reports (mainly autopsy studies) detected no malignant cells in ovarian tissue from patients affected by colorectal cancer, but on the contrary the possible ovarian involvement by rectal cancer is well described and therefore no definitive conclusions can be drawn concerning safety and efficacy of fertility preservation procedures for such women yet [4].

## 6. Future Perspectives

### 6.1. In Vitro Maturation (IVM)

IVM of immature ovarian follicles collected from ovarian tissue at the time of cryopreservation could be a promising option for patients affected with types of cancer at high risk of ovarian localization, which should be advised against ovarian tissue cryopreservation and subsequent grafting for the high risk of relapse [47].

This technique consists in the aspiration of immature oocytes from preantral and small antral ovarian follicles present in ovarian tissue before or after ovarian tissue withdrawal and the subsequent incubation in a maturation medium, with final vitrification of mature oocytes or cryopreservation of embryos produced with IVF [66].

This method can be applied also in prepubertal girls, independently from cycle phase, and allow subsequent IVF and pregnancy, decreasing the risk of reimplantation of malignant cells. IVM is currently adopted in many ART centers with a 20 to 35% live birth rate from cryopreserved IVM oocytes, but more research is needed regarding the application in the specific field of fertility preservation to improve cryopreservation protocols and culture media [4, 66–69].

### 6.2. “Artificial Ovary”

Great interest has been awakening in recent years for the possibility of creating an “artificial ovary”, with the objective of reducing the possibility of disseminating malignant cells and maximizing the chances of survival and growth of isolated follicles and ovarian cells, recreating their optimal microenvironment [70–73].

The concept of the “artificial ovary” consists in the packaging of oocytes and follicular cells in a biodegradable scaffold to maintain the three-dimensional structure. The scaffold should be constituted of a matrix to maintain the interactions between the oocyte and granulosa cells, in the while revascularization occurs. In 2012 Vanacker and colleagues first experimented a biodegradable matrix of alginate hydrogel containing isolated follicles and ovarian cells, transplanted in a peritoneal pocket in immunocompetent mice [73]. Further research which focused on the study of other materials is needed to increase the survival of the grafting by reducing the damage resulting from ischemia and oxidative stress [72].

## 7. Conclusions

The progresses in oncologic treatments with consequently more women affected by iatrogenic POI as well as the postponing of childbearing in women, with consequent low chances of pregnancy due to scarce ovarian reserve, are raising interest concerning the possibility of preserving fertility.

In its most recent guidelines ASCO has clarified the requirement to address all the women requiring potentially gonadotoxic treatments to fertility preservation options, irrespective of age, parity, or prognosis [22].

At present oocyte and embryo cryopreservation are the main fertility preservation options for postpuberal women who can delay treatments for 10-15 days, as they are the only techniques approved as fertility preservation options at the moment. Nevertheless, patients undergoing COS for fertility preservation are generally not infertile women; therefore they can be easily responsive also to mild gonadotrophins stimulation schedules.

On the other hand, ovarian tissue cryopreservation is currently the only option for prepuberal girls or patients who require immediate treatments, but it is still considered an experimental technique until more research will assure its complete safety.

Finally, to increase the chances of success, in the future a combination of techniques could probably be adopted, for example, by administering COS before oocyte aspiration and subsequent ovarian tissue withdrawal and cryopreservation. This procedure could allow cryopreservation of both IVM oocytes and ovarian tissue, also for isolated follicles and ovarian cells grafting, thus potentially minimizing the risk of neoplastic dissemination.

## Figures and Tables

**Table 1:** Options for fertility preservation (FP).

**FP option**	**Pros**	**Cons**
**GnRH agonists co-administration during oncologic treatments**	(i) Some medical benefits	(i) Uncertain efficacy as FP option (ii) Potential relevant side effects

**Oophoropexy**	(i) Relatively simple and quick laparoscopic surgical procedure	(i) Useful only in patients undergoing pelvic radiotherapy

**Embryo cryopreservation**	(i) Well established and reliable FP technique (ii) No risk of reimplanting malignant cells	(i) Only feasible in post-puberal women with a male partner (ii) Requires time for COS (iii) Does not restore fertility but only chance of pregnancy

**Oocyte cryopreservation**	(i) Well established and reliable FP technique (ii) Also feasible in single women (iii) No risk of reimplanting malignant cells	(i) Requires time for COS (ii) Does not restore fertility but only chance of pregnancy (iii) Only feasible in post-puberal women

**Ovarian tissue cryopreservation and grafting**	(i) Also feasible in pre-puberal girls (ii) Do not delay oncologic treatments (iii) Restore ovarian function	(i) Age limitations (<35-36 yrs) (ii) Invasive surgical procedure under general anesthesia (iii) Considered experimental as FP option at present
